# Real-Time Detection of Orbital Maneuvers Using Epoch-Differenced Carrier Phase Observations and Broadcast Ephemeris Data: A Case Study of the BDS Dataset

**DOI:** 10.3390/s20164584

**Published:** 2020-08-15

**Authors:** Rui Tu, Rui Zhang, Lihong Fan, Junqiang Han, Pengfei Zhang, Xiaochun Lu

**Affiliations:** 1 National Time Service Center, Chinese Academy of Sciences, Shu Yuan Road, Xi’an 710600, China; zhangrui@ntsc.ac.cn (R.Z.); fanlihong@ntsc.ac.cn (L.F.); hanjunqiang@ntsc.ac.cn (J.H.); zhangpengfei@ntsc.ac.cn (P.Z.); luxc@ntsc.ac.cn (X.L.); 2 Key Laboratory of Time and Frequency Primary Standards, Chinese Academy of Sciences, Xi’an 710600, China; 3 School of Astronomy and Space Science, University of Chinese Academy of Sciences, Yu Quan Road, Beijing 100049, China

**Keywords:** BDS, orbital maneuvers, carrier phase observation, velocity estimation, epoch-difference

## Abstract

The orbital maneuvers of the global navigation satellite system (GNSSs) have a significant influence on the performance of the precise positioning, navigation, and timing (PNT) services. Because the Chinese BeiDou Navigation Satellite System (BDS) has three types of satellites in the geostationary orbit (GEO), inclined geosynchronous orbit (IGSO), and medium earth orbit (MEO) maneuvers occur more frequently. Thus, it is essential to determine an effective approach for the detection of orbital maneuvers. This study proposes a method for the detection of orbital maneuvers using epoch-differenced carrier phase observations and broadcast ephemeris data. When using the epoch-differenced velocity estimation as a basic data solution model, the time discrimination and satellite identification factors are defined and used for the real-time detection of the beginning and the pseudorandom noise code (PRN) of satellites. The datasets from four GNSS stations (WUH1, BJF1, POHN, CUT0) from the year 2016 were collected and analyzed. The validations showed that the beginning, the PRN of the orbital maneuver of the satellite can be precisely detected in real time for all GEO, IGSO, and MEO satellites, and the detected results also showed good consistency, with the beginning time at a difference of 1–2 min across different stations. The proposed approach was observed to be more sensitive, and the detected beginning time was about 30 min earlier than the single point positioning approach when the high-precision carrier phase observation was used. Thus, orbital maneuvering can be accurately detected by the proposed method. It not only improves the utilization of the collected data but also improves the performance of PNT services.

## 1. Introduction

The BeiDou Navigation Satellite System (BDS) was developed and constructed independently by China, and can provide precise positioning, navigation, and timing (PNT) services. The fully constructed system comprises three stages: BeiDou navigation satellite demonstration system (BDS-1), BeiDou regional navigation satellite system (BDS-2), and BeiDou global navigation satellite system (BDS-3) [1,2]. Since December 27, 2012, BDS-2 has been providing continuous PNT services across the Asia–Pacific region with five geostationary orbit (GEO) satellites, five inclined geosynchronous orbit (IGSO) satellites, and four medium earth orbit (MEO) satellites. Currently, there are seven IGSO satellites and three MEO satellites in the BDS-2 [3]. The BDS-3 global networking started in November 2017, and finished in June 2020, it began to provide global service for users at the end of July in 2020. Right now, the space segment of BDS-3 consists of 30 satellites, which include 3 GEO, 3 IGSO, and 24 MEO satellites [4].

In general, the non-spherical gravity of the Earth and other perceptual factors affect the navigation satellites and lead to long-term perturbations of orbital elements and the offset of the satellite location. Thus, orbital maneuvering is required to keep the navigation satellites in the nominal orbital position, especially for the GEO and IGSO satellites as the orbital maneuver is more frequent [5]. The propulsion system is usually employed to change the orbit of a satellite. Unlike other global navigation satellite system (GNSS) systems, such as global positioning system (GPS), GLObalnaya NAvigatsionnaya Sputnikovaya Sistema (GLONASS), and Galileo, in the BDS with hybrid constellations, which does not include the MEO but contains both the GEO and IGSO, the orbital maneuver is more frequent. During orbital maneuvering, satellite positions vary by tens of kilometers to the normal orbit position, causing serious impacts on its functioning as well as additional positioning, and orbit determination needs to be performed [6]. To adjust the strategies for positioning and orbit determination in a timely manner, the abnormal condition and maneuvers must be determined as soon as possible after they occur [7]. In practice, satellite maneuver information for the BDS is not available in real time, and only the hourly ephemeris broadcasts provide the satellite’s health status, which contains the maneuver information. Owing to the low time sampling rate and identification errors of satellite abnormity of the BDS broadcast ephemeris, users may lose plenty of effective and useful observation data, especially true when observation conditions are bad [8]. Thus, developing a real-time detection approach for satellite orbital maneuvers with high time resolution and reliability is important and valuable.

Recently, many researchers have proposed methods for the detection of orbital maneuvers. Pervan et al. detected the GPS orbit errors using short-baseline carrier-phase measurements [9,10] and used them for the integrity service. Scire et al. analyzed the spatial debris orbit determination algorithm without the orbital maneuver detection by the space-based optical observation data [11]. Cui et al. used orbital residuals and the force model to determine the orbital maneuvers of objects in space [12], but this needs a complex force model. Du et al. used orbital monitoring data from the China Area Positioning System (CAPS) to determine the orbital maneuvers of the GEO satellites [13]; however, this is not suitable for typical users. Su et al. used the mechanical energy difference between the spacecraft and space targets to detect the orbital maneuvering, but the effectiveness of this method was restricted by the number of stations [14]. Ye et al. used the mutual difference from the orbits, before and after the maneuvering, to detect the orbit maneuvering of the BeiDou GEO and IGSO satellites [15], but the detection was not executed in real time. Huang et al. used the residuals of single point positioning (SPP) to detect the orbital maneuvering in real time, but only used low-precision pseudorange observations [16]. In addition, Qiao et al. studied the Beidou satellite maneuver thrust force estimation, and Dai et al. analyzed the precise orbit determination for the GNSS maneuvering satellite [17].

Based on this background, this work proposes an approach for the determination of orbital maneuvers in real time using high-precision carrier phase observations by epoch-differenced velocity estimation, validated by BDS data. The proposed method can be operated in real time by a station-alone receiver and does not need any additional data information or the orbit force mode. As it uses high-precision carrier phase observations, the detection of orbital maneuvers is more sensitive and rapid.

This work first introduces the principle of epoch-differenced velocity estimation and then establishes the method for the detection of orbital maneuvers and determination of the pseudorandom noise code (PRN) of maneuverable satellites. Finally, the approach is validated and applied for BDS datasets.

## 2. Algorithms

Epoch-differenced observations of GNSS can directly estimate the velocity of the station by the broadcast ephemeris. For a static station, the velocity should be zero, if there is no orbital maneuver. During an orbital maneuver, however, due to errors in the reading of the broadcast ephemeris, the estimated velocity will be abnormal, and the observation residuals will exhibit large mutations. Thus, velocity estimations can be used for the detection of orbital maneuvers.

In this section, the algorithm of the epoch-differenced velocity estimation using the GNSS carrier phase observation is first introduced, and then the details of the method for the detection of the start time of the orbital maneuvers and the corresponding PRN determination methods are presented.

### 2.1. Epoch-Differenced Velocity Estimation

For the raw carrier phase observations, the epoch-differenced velocity estimation can be expressed as [18,19]

(1) $${\lambda \Phi }_{r}^{s}=\rho_{r}^{s}+c\left(\delta T_{r}-\delta T^{s}\right)+T_{r}^{s}-I_{r}^{s}-\lambda N_{r}^{s}+M_{r}^{s}+U_{r}^{s}+\varepsilon_{r}^{s}$$

where subscript *s* refers to a satellite and *r* refers to a receiver; λ is the carrier phase wavelength; *c* is the speed of light; $\Phi_{r}^{s}$ is the carrier phase observation of the receiver with respect to the satellite; $\rho_{r}^{s}$ is the geometric distance from the satellite to the receiver; $\delta T_{r}$ and $\delta T^{s}$ are the receiver and satellite clock errors, respectively; $T_{r}^{s}$ and $I_{r}^{s}$ are the tropospheric and ionospheric delays, respectively; $N_{r}^{s}$ is the carrier phase ambiguity; $M_{r}^{s}$ is the sum of the modeling errors, such as solid Earth tide and ocean loading, relativistic effects, phase center offset, and phase wind-up; $U_{r}^{s}$ represents the un-modeling errors, such as ephemeris residual, atmospheric residual, and multipath effects; and $\varepsilon_{r}^{s}$ is the measurement noise [20,21].

As the dual-frequency GNSS observations used are free from cycle slips, the first-order ionospheric term can be canceled by applying the ionospheric-free combination [22]. The differenced equation between two consecutive epochs (t, t+1) can then be written as follows:

(2) $$\begin{array}{ll} \alpha {\left[{\lambda \Delta \Phi }_{r}^{s}\left(t,\;t+1\right)\right]}_{L_{1}} & +\beta {\left[{\lambda \Delta \Phi }_{r}^{s}\left(t,\;t+1\right)\right]}_{L_{2}}\\ & ={\Delta \rho }_{r}^{s}\left(t,\;t+1\right)+c\left[\delta T_{r}\left(t,t+1\right)-\delta T^{s}\left(t,t+1\right)\right]+\Delta T_{r}^{s}\left(t,t+1\right)\\ & +M_{r}^{s}\left(t,t+1\right)+U_{r}^{s}\left(t,t+1\right)+\varepsilon_{r}^{s}\left(t,t+1\right) \end{array}$$

where $\alpha =f_{L_{1}}^{2}/\left(f_{L_{1}}^{2}-f_{L_{2}}^{2}\right)$ and $\beta =-f_{L_{2}}^{2}/\left(f_{L_{1}}^{2}-f_{L_{2}}^{2}\right)$ are the standard coefficients of the ionosphere-free combination and Δ represents the time single-difference. It is to be noted that the value of t represents the time of the observation in units equal to the observation sample interval. The term ${\Delta \rho }_{r}^{s}\left(t,\;t+1\right)$ usually depends upon the change in the geometric range due to the satellite’s orbital motion, station’s motion, and the Earth’s rotation, as well as factors in the variation of the solid Earth tide and ocean loading [23]. When the station is static, the Earth’s rotation, solid Earth tide, and ocean loading are corrected using the proper model, thus, the term ${\Delta \rho }_{r}^{s}\left(t,\;t+1\right)$ directly responds to the satellite’s orbital motion, which can be considered as a 3D displacement ${\Delta \xi }_{r}\left(t,\;t+1\right)$ in an Earth-centered Earth-fixed (ECEF) reference frame during the interval (t, t+1). Here, it should be noted that the displacement term ${\Delta \xi }_{r}\left(t,\;t+1\right)$ can be regarded as the velocity over the interval (t, t+1) itself [19].

Meanwhile, the troposphere delay can be corrected with precise modeling, and the satellite clock errors can be corrected using the broadcast ephemeris [24]. The corrected residual can also be weakened by the epoch difference of two adjacent epochs; thus, the final equation can be rewritten as follows:

(3) $$\alpha {\left[{\lambda \Delta \Phi }_{r}^{s}\left(t,\;t+1\right)\right]}_{L_{1}}+\beta {\left[{\lambda \Delta \Phi }_{r}^{s}\left(t,\;t+1\right)\right]}_{L_{2}}=e_{r}^{s}\cdot {\Delta \xi }_{r}\left(t,\;t+1\right)+\;c\delta T_{r}\left(t,t+1\right)+\;U_{r}^{s}\left(t,t+1\right)+\varepsilon_{r}^{s}\left(t,t+1\right)$$

where $\alpha {\left[{\lambda \Delta \Phi }_{r}^{s}\left(t,\;t+1\right)\right]}_{L_{1}}+\beta {\left[{\lambda \Delta \Phi }_{r}^{s}\left(t,\;t+1\right)\right]}_{L_{2}}$ are the time single-difference ionosphere-free observations, $e_{r}^{s}$ is the unit vector from the satellite to the receiver at epoch, and the symbol ⋅ indicates the scalar product between the vectors $e_{r}^{s}$ and ${\Delta \xi }_{r}\left(t,\;t+1\right)$. The unknown parameters are the 3D velocity ${\Delta \xi }_{r}\left(t,\;t+1\right)$ and the receiver clock error variation $\delta t_{r}\left(t,t+1\right)$. The un-modeled error $U_{r}^{s}\left(t,t+1\right)$ is not considered and will be absorbed by the estimated velocity. $\varepsilon_{r}^{s}\left(t,t+1\right)$ is the noise term, as described previously.

The observation weight of the stochastic model can be expressed as follows [25,26]:

(4) $$p=\{\begin{array}{cc} 1 & \;\theta >30{}^{\circ}\\ 2\sin\left(\theta \right) & \;\theta \leq 30{}^{\circ} \end{array}$$

where p represents the observed weight and θ is the average satellite elevation of the two adjacent epochs. Based on Equations (3) and (4), the least-squares method can be employed for velocity estimation to track at least four satellites for two generic consecutive epochs. Here, it is useful to note that the observations should be free from cycle slips for the adjacent epochs.

### 2.2. Detection of the Orbital Maneuvers

The mean square error of unit weight (MSE) can be calculated by the least-squares method [16].

(5) $$V=A\hat{x}-\;L$$

(6) $$\hat{\sigma_{0}}\left(t,t+1\right)=\sqrt{V^{T}PV/\left(n-m\right)},\;(n>m)$$

where V is the observation residual, A is the designed coefficient matrix for the estimated parameters, $\hat{x}$ is the estimated parameter, L is the constant term estimated by the observations after correction, and n and m are the numbers of observations and estimated parameters, respectively.

The detection of the satellite orbital maneuver can be described as follows:

(7) $$\sigma_{M}\left(t,t+1\right)=\hat{\sigma_{0}}\left(t,t+1\right)-3M_{healthy}$$

where $\sigma_{M}\left(t,t+1\right)$ is the time discriminant factor and $M_{healthy}$ is the empirical threshold determined by the root mean squares (RMS) of the $\hat{\sigma_{0}}$ time series during the healthy period of the satellites. When $\sigma_{M}\left(t,t+1\right)$ is greater than zero and lasts for 5 min, the corresponding time is considered as the start time of the orbital maneuver.

In addition, as the test results show that the standard deviation (STD) of the observation residual is more sensitive than the MSE, we used the STD of the observation residual for the detection of orbital maneuvers in this study.

(8) $$S_{M}\left(t,t+1\right)=STD_{M}\left(t,t+1\right)-3S_{healthy}$$

where $S_{M}\left(t,t+1\right)$ is the new time discriminant factor. $S_{healthy}$ is the empirical threshold, which is determined by the STD of the observation residual time series during the healthy period of the satellite. When $S_{M}\left(t,t+1\right)$ is greater than zero and lasts for 5 min, the corresponding time is considered as the start time of the orbital maneuver.

### 2.3. Determine the PRN of the Maneuverable Satellites

The start time of the orbital maneuver can be detected by the MSE or the STD of the observation residual. Moreover, we need to determine the PRN number of the maneuvering satellites. In this study, the array L was used to detect the PRN of the maneuvering satellite. From Equation (2), L can be calculated by the following equations:

(9) $$L=\alpha {\left[{\lambda \Delta \Phi }_{r}^{s}\left(t,\;t+1\right)\right]}_{L_{1}}+\beta {\left[{\lambda \Delta \Phi }_{r}^{s}\left(t,\;t+1\right)\right]}_{L_{2}}-{\Delta \rho }_{r}^{s}{}_{0}\left(t,\;t+1\right)-\delta T^{s}\left(t,t+1\right)]\;-\Delta T_{r}^{s}\left(t,t+1\right)-M_{r}^{s}\left(t,t+1\right)-U_{r}^{s}\left(t,t+1\right).$$

The term ${\Delta \rho }_{r}^{s}{}_{0}\left(t,\;t+1\right)$ represents the initial value of the term ${\Delta \rho }_{r}^{s}\left(t,\;t+1\right)$ and is calculated by the satellite position and station position. During the orbital maneuvering period, the broadcast ephemeris is no longer correct; thus, the calculated L would have a gross error. Thus, it can be used to detect which satellite is maneuvering. It is to be noted that L also contains the term for receiver clock variation as the initial receiver clock error is not known. To eliminate the influence of the receiver clock, we employ a single-difference between different satellites.

Thus, the new L can be written as follows:

(10) $$L^{k,j}=\left|L^{k}-L^{j}\right|$$

where $L^{k,j}$ is the satellite identification factor calculated by the satellite-differenced L value between the satellite k and reference satellite j, and | | indicates the absolute value. The reference satellite j should be healthy. If the selected reference satellite j is unhealthy (with orbital maneuver), all the $L^{k,j}$ values calculated will show a jump and have a much larger value than the former values; thus it is easy to exclude the reference satellite with orbital maneuver. When the orbital maneuver has been detected, the PRN of the orbital maneuver satellite can be defined as the satellite j corresponding to the largest $L^{k,j}$ value.

## 3. Validation

To validate the proposed approach, first, an example of orbital maneuver detection by this method is provided. It contains the datasets, comparison of the sensibility of orbital maneuver detection and PRN determination by different methods, performance analysis of the consistency by different stations, and the analysis of the effectiveness of SPP and velocity estimation. Then, the method was applied for the GEO, IGSO, and MEO satellites.

### 3.1. Datasets

**Figure 1:**
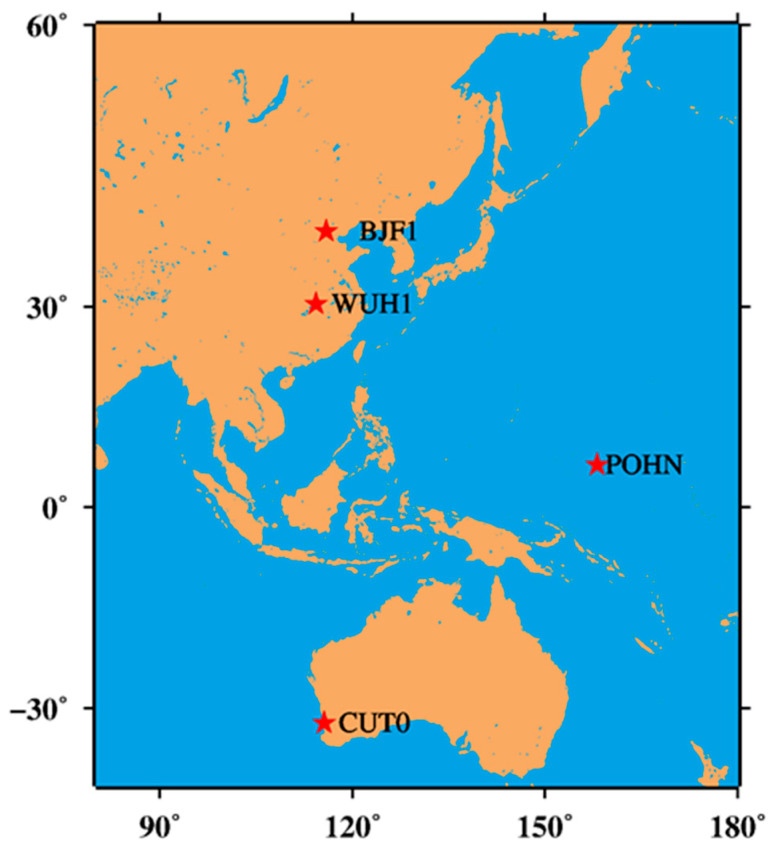
Distribution of the four selected global navigation satellite system (GNSS) stations.

To test the feasibility of the proposed approach, four international GNSS stations were selected for validation in the year 2016, and the sampling rate was 30 s. Figure 1 shows the distribution of the four stations, namely, BJF1, WUH1, POHN, and CUT0, which could track more than four BDS satellites during the test period. The details of the stations are shown in Table 1. The broadcast ephemeris data are obtained from the GeoForschungsZentrum Potsdam (GFZ) multi-GNSS orbit product (indicated by GBM) and the earth rotation parameters (ERP), satellite phase center offset (PCO), and variation (PCV) are provided by Center for Orbit Determination in Europe, Switzerland (CODE) [27]. The initial coordinates of the stations were estimated by the weekly precise point positioning (PPP), with a precision of 1–2 cm.

### 3.2. Detection of the Orbital Maneuvers

First, the data from the CUT0 station obtained on July 18, 2016 was used. On this day, the BDS GEO satellites C04 exhibited an orbital maneuver from UTC time 07:00:00 to 13:00:00, which was marked by the broadcast ephemeris.

**Figure 2:**
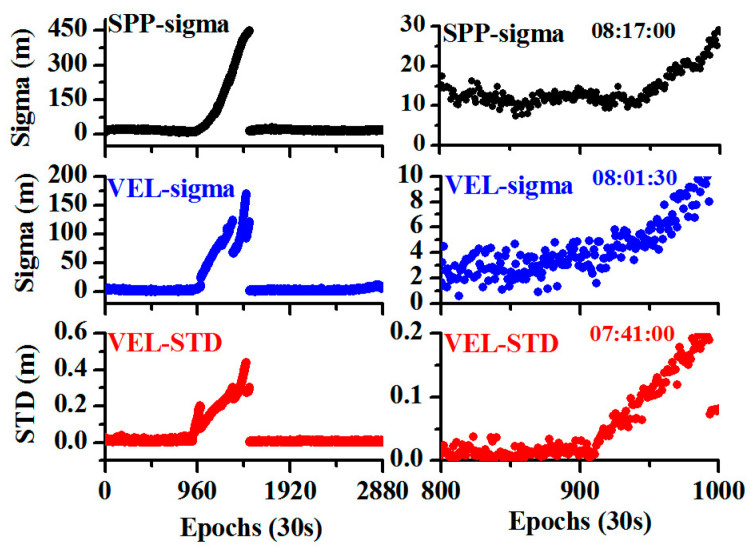
Comparison of different approaches for the detection of the orbital maneuver (the black color represents the mean square error (MSE) for single point positioning (SPP), the blue color represents the MSE for velocity estimation, and the red color represents the standard deviation STD for velocity estimation; the left plots denote the values of estimated parameters during one day, and the right plots denote the values of the estimated parameters during the maneuver period).

As both the SPP and velocity estimations could be used for the detection of the orbital maneuver, we compared them. From Figure 2, it can be seen that the MSE time series increased after the orbital maneuver started, irrespective of the method of estimation. Thus, orbital maneuvers can be detected even when the test values exceed the threshold values over time.

In general, while the orbital maneuver begins, the precise orbit cannot be determined as the orbital maneuver leads to the failure of the kinetic empirical parameters. Thus, a fast and precise judgment of the beginning of the orbital maneuver is important for orbit determination. The beginning, determined using the broadcast ephemeris, is usually one or two hours earlier than the real orbital maneuver, thus, one or two hours worth of data for precise orbit determination is lost. Figure 2 shows that the MSE of the SPP and velocity estimation have a large amount of noise, and the methods are not very sensitive. The time detected by SPP is 08:17:00 and that by velocity estimation is 08:01:30. Thus, we used the observation residual to detect the beginning of orbital maneuver. As also shown in Figure 2, the STD of the observation residual is more sensitive and can detect the beginning more rapidly; the detected time is 07:41:00.

**Figure 3:**
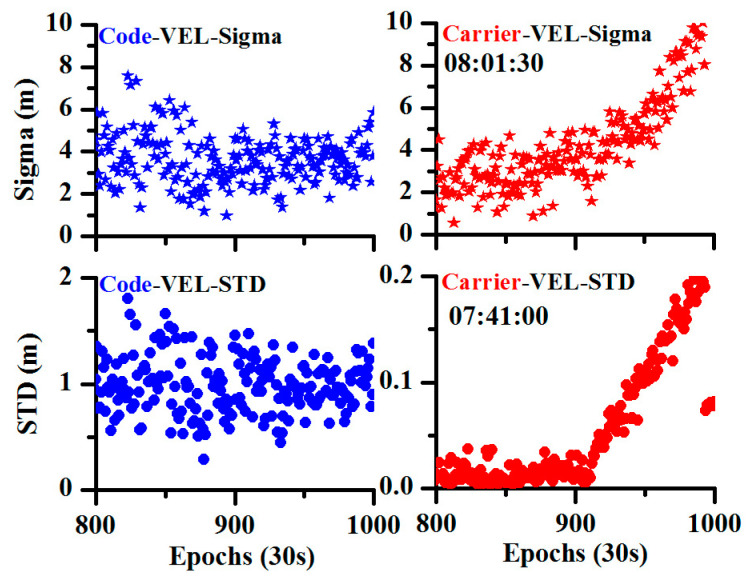
Comparison of different observations for the detection of the orbital maneuver (the blue color represents pseudorange observations, and the red color represents carrier phase observations).

In addition, as the velocity estimation can be operated by a single pseudorange or single carrier phase observation, Figure 3 shows the comparison between the MSE and STD of the observation residual for the pseudorange and carrier phase observations. It can be concluded that whether for the MSE or the STD, the carrier phase observations were more sensitive due to their high precision.

### 3.3. Determination of the PRN of the Maneuverable Satellites

**Figure 4:**
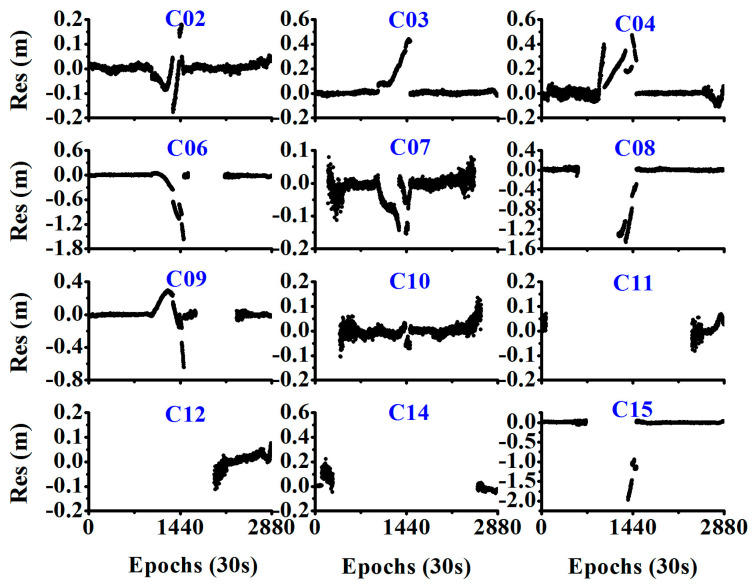
Observation residuals of different PRN satellites (Satellite C04 showed orbital maneuver from 07:00:00 to 13:00:00 as determined from the broadcast ephemeris).

While the orbital maneuver is detected by the MSE or the STD of the observation residual, we also need to determine the PRN of the maneuverable satellites. During the period of the orbital maneuver, if the wrong satellite positions are used, the estimated velocity will have a bias, and the observation residual will be abnormal. As shown in Figure 4, it is difficult to determine which PRN is the true for the maneuvering satellite as all the observation residuals show abnormal values during the maneuver period.

**Figure 5:**
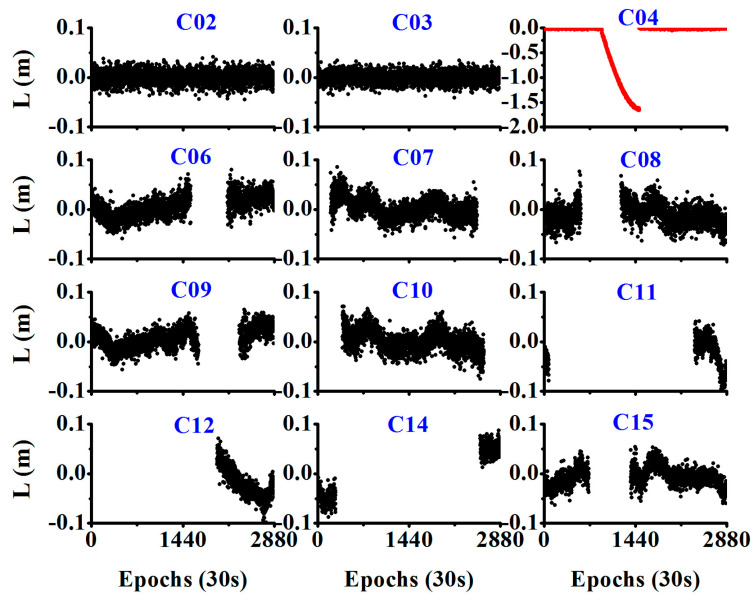
Time series of the array L for different PRNs during the orbital maneuver period (C01 was selected as the reference satellite, and C04 was the orbit maneuvering satellite).

In this study, the satellite-differenced L value between the non-reference satellite and reference satellite is used to detect which PRN is the maneuverable satellite. As shown in Figure 5, for the healthy satellites, the satellite-differenced L value was usually smaller than 0.05 m, and for the orbit maneuvering satellite, it showed very large values and increased rapidly, as shown in red in Figure 5.

## 4. Discussion

### 4.1. Performance Analysis of the Consistency

**Figure 6:**
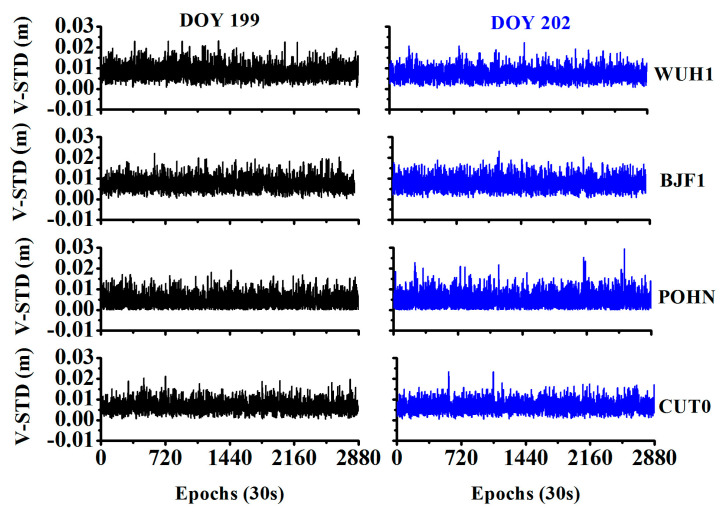
STD time series of the observation residuals for different stations and different days.

As we used the STD of the observation residuals to detect the orbital maneuver, it is important to analyze the stability and consistency of the STD time series of the observation residuals. Figure 6 shows that the STD of the observation residuals by velocity estimation was stable over different days and was also consistent across different stations.

Table 2 provides the statistics for the STD of the observation residuals during the non-maneuvering period. The mean values are (0.0082, 0.0075, 0.0044, 0.0067) m and the corresponding STDs are (0.0036, 0.0033, 0.0033, 0.0031) m for day of year (DOY) 199. Further, the mean values are (0.0077, 0.0077, 0.0049, 0.0066) m and corresponding STDs are (0.0031, 0.0034, 0.0037, 0.0030) m for DOY 202 at the stations WUH1, BJF1, PONH, and CUT0, respectively. The final time discriminant factors are determined as (0.026, 0.025, 0.017, 0.022) m for these corresponding stations.

**Figure 7:**
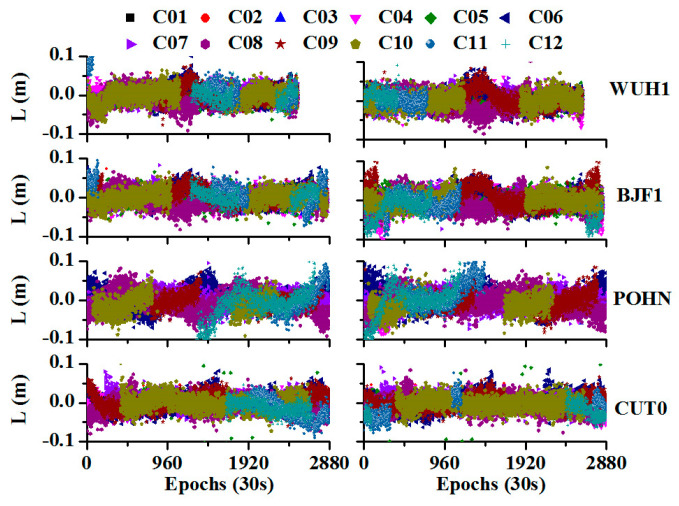
Time series of the array L for different stations and different days (the left side represents the day of year (DOY) 199, and the right side represents the DOY 202).

Figure 7 shows the time series of the array L for different satellites and different days during the non-maneuvering period. Clearly, L for all of the satellites is stable for different days and shows a normal distribution. It is also consistent across different stations.

Table 3 shows the statistics of the L array during the non-maneuvering period, the mean values are (−0.0002, 0.0001, −0.0002, 0.0003) m and the corresponding STDs are (0.018, 0.016, 0.022, 0.015) m for DOY 199, and the mean values are (0.0002, −0.0001, 0.0001, −0.0002) m and the corresponding STDs are (0.021, 0.024, 0.023, 0.016) m for DOY 202 at the stations WUH1, BJF1, PONH, and CUT0, respectively.

**Figure 8:**
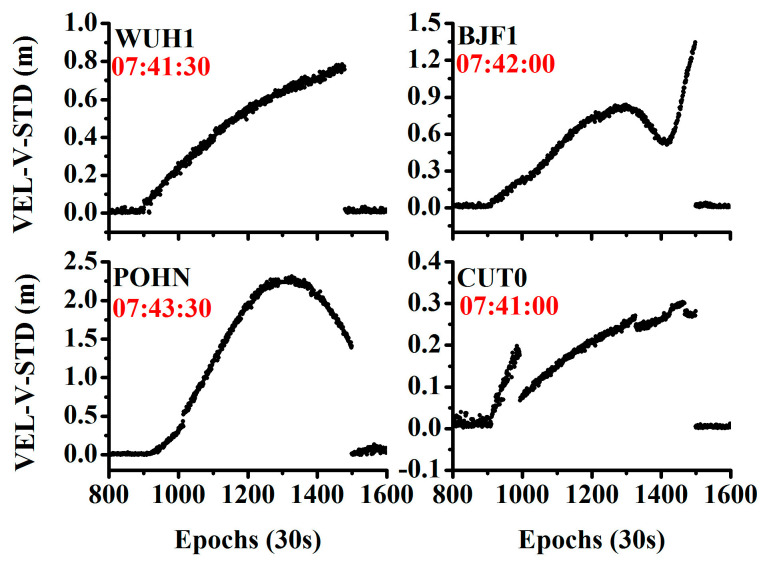
Time series of the STD of the observation residuals for different stations on 18 July 2016.

In addition, Figure 8 shows the time series of the time discriminant factor at four different stations on July 18, 2016, where C04 is the satellite showing orbital maneuver. Evidently, the time discriminant factor changed rapidly and was larger than the threshold after the start of the true orbital maneuver. It returned to the normal value after the orbital maneuver ended. The detected beginning times were (07:41:30, 07:42:00, 07:43:30, 07:41:00) at the stations WUH1, BJF1, POHN, and CUT0, respectively.

**Figure 9:**
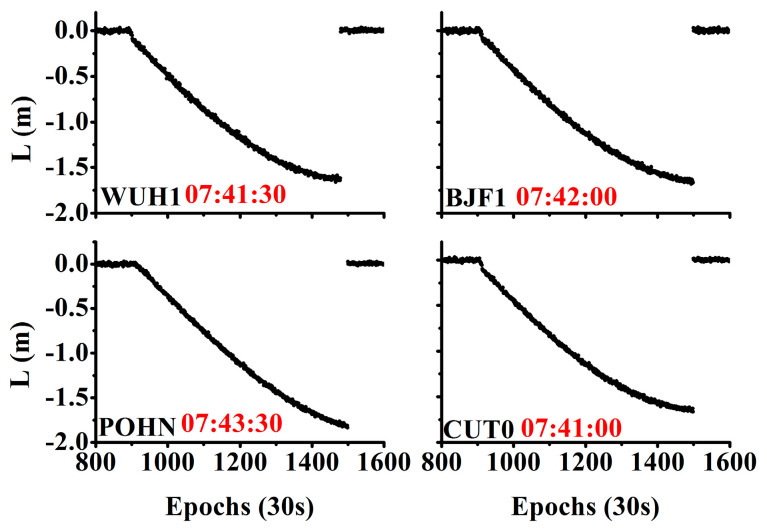
Time series of the array L for satellite C04 at different stations on 18 July 2016.

Figure 9 shows the time series of the array L for the satellite C04 at different stations on July 18, 2016. When the orbital maneuver began, the array L increased rapidly, was consistent across different stations, and returned to the normal values when the orbital maneuver ended.

**Figure 10:**
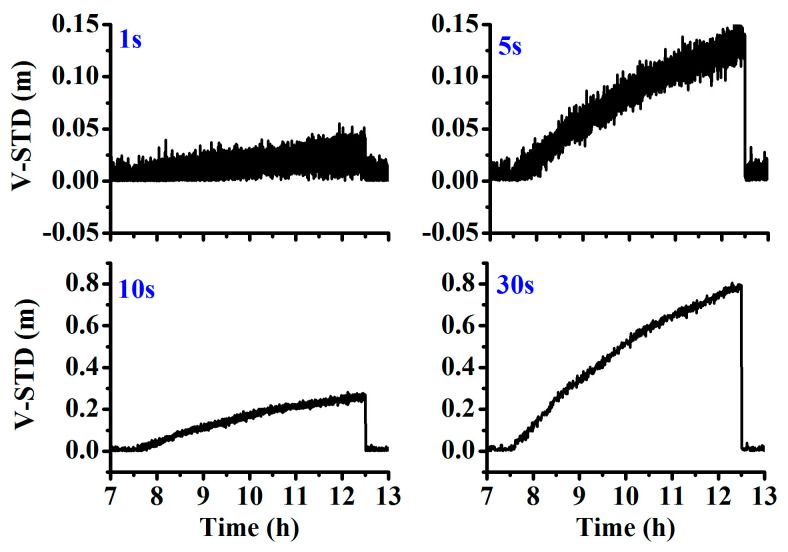
STD of the observation residuals for different sample rates at the station CUT0.

While high-rate GNSS is widely used for real-time applications, we also tested the performance of the different sampling rates for the orbital maneuver detection. As shown in Figure 10, with the increase in the sample rate, the calculated time discriminant factor exhibits a high amount of noise. When the sample rate is larger than 0.2 Hz (5 s), it consists almost entirely of noise signals; thus high-rate observations are not suggested for use in orbital maneuver detection.

### 4.2. Analysis of the Effectiveness of the Method

**Figure 11:**
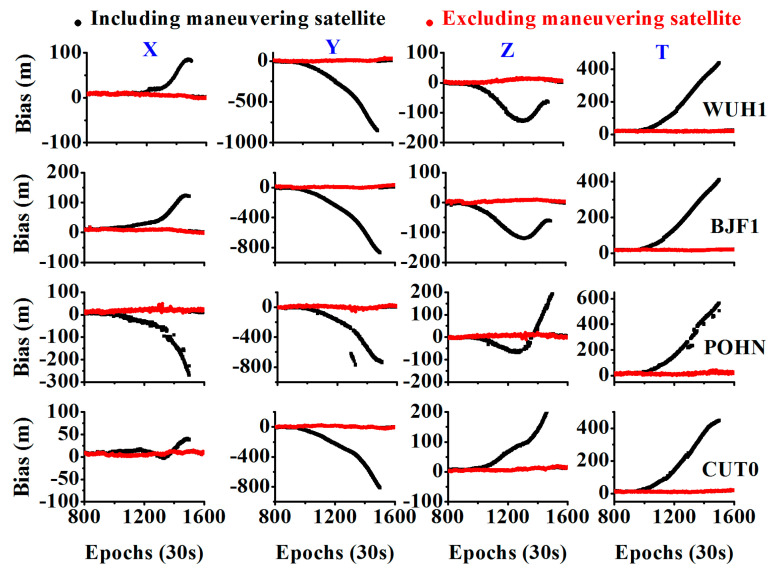
Comparison between the SPP results when including the maneuvering satellite and excluding the maneuvering satellite.

To further validate the effectiveness of the proposed method, we first used the SPP results to evaluate it. As shown in Figure 11, when including the maneuvering satellite, the position bias deviates from the value of zero and increases to a hundred or a thousand meters. In addition, the estimated clock error shows a large bias, and its value increases. When excluding the maneuvering satellite, the position bias and clock error are set to zero. It is to be noted that the clock error time series subtracted the value of the first epoch for the picture drawing.

**Figure 12:**
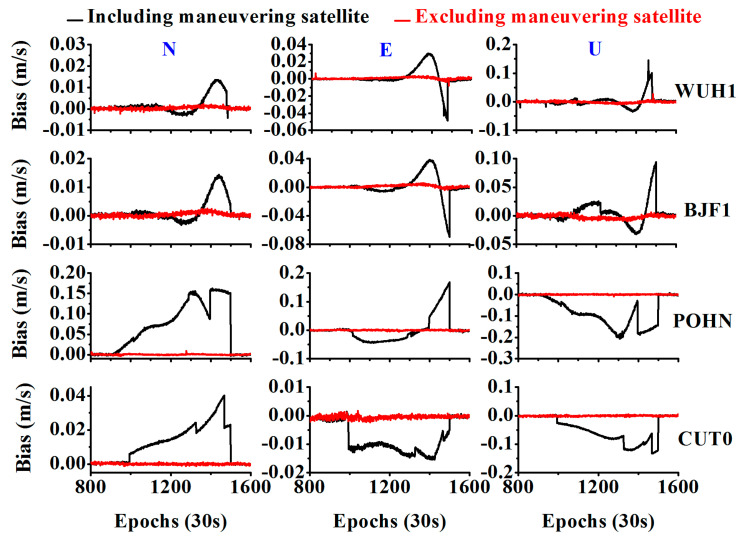
Comparison between the velocity results when including the maneuvering satellite and excluding the maneuvering satellite.

In addition, we used velocity estimations to validate the effectiveness of the method. As shown in Figure 12, when including the maneuvering satellite, the velocity deviates from the value of zero, and when excluding the maneuvering satellite, the velocity returned to zero.

**Figure 13:**
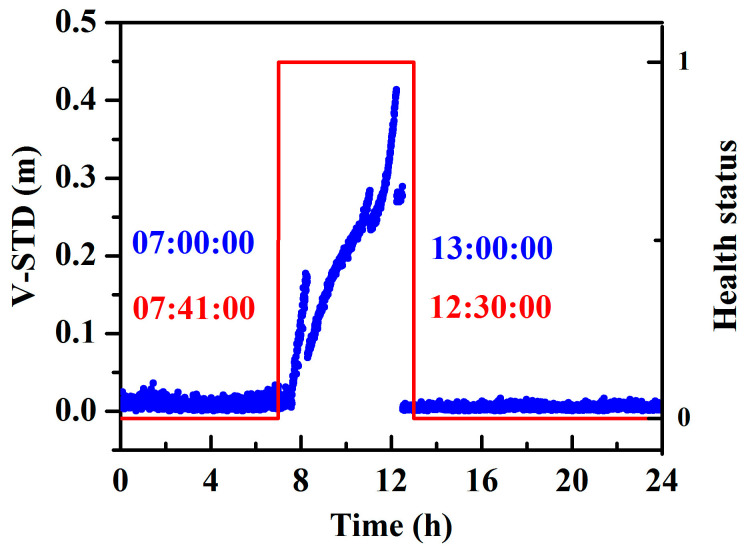
Comparison between the marked time of orbital maneuver by the broadcast ephemeris and real-time detection approach (health status: “0” represents healthy, and “1” represents unhealthy).

As the broadcast ephemeris also marked the beginning and the ending of the orbital maneuver, in Figure 13, the marked time of orbital maneuver determined by the broadcast ephemeris and real-time detection approach are compared. It can be seen that the detected time window (from 07:41:00 to 12:30:00) falls within the time window determined from the broadcast ephemeris (from 07:00:00 to 13:00:00).

### 4.3. Application to Different Constellations

**Figure 14:**
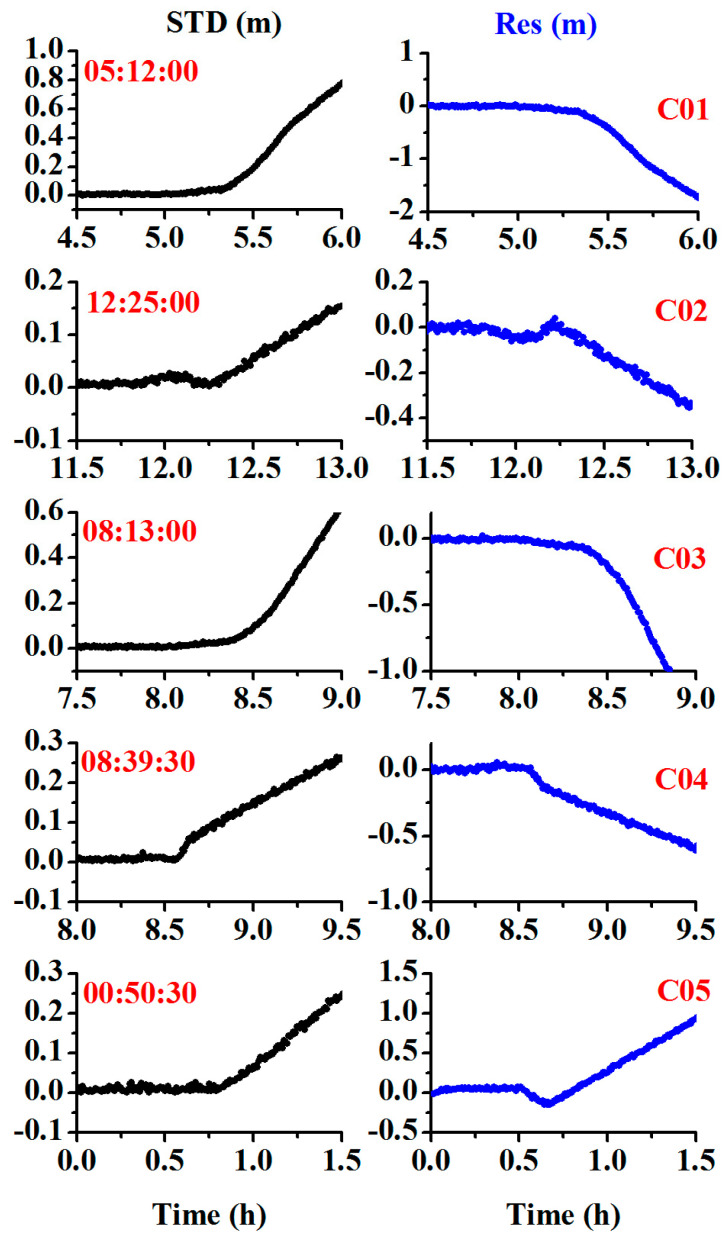
Time series of the STD of the observation residuals and array L for the geostationary orbit (GEO) satellites showing orbital maneuver (the left side represents the time discriminant factor, and the right side represents the satellite identification factor).

**Figure 15:**
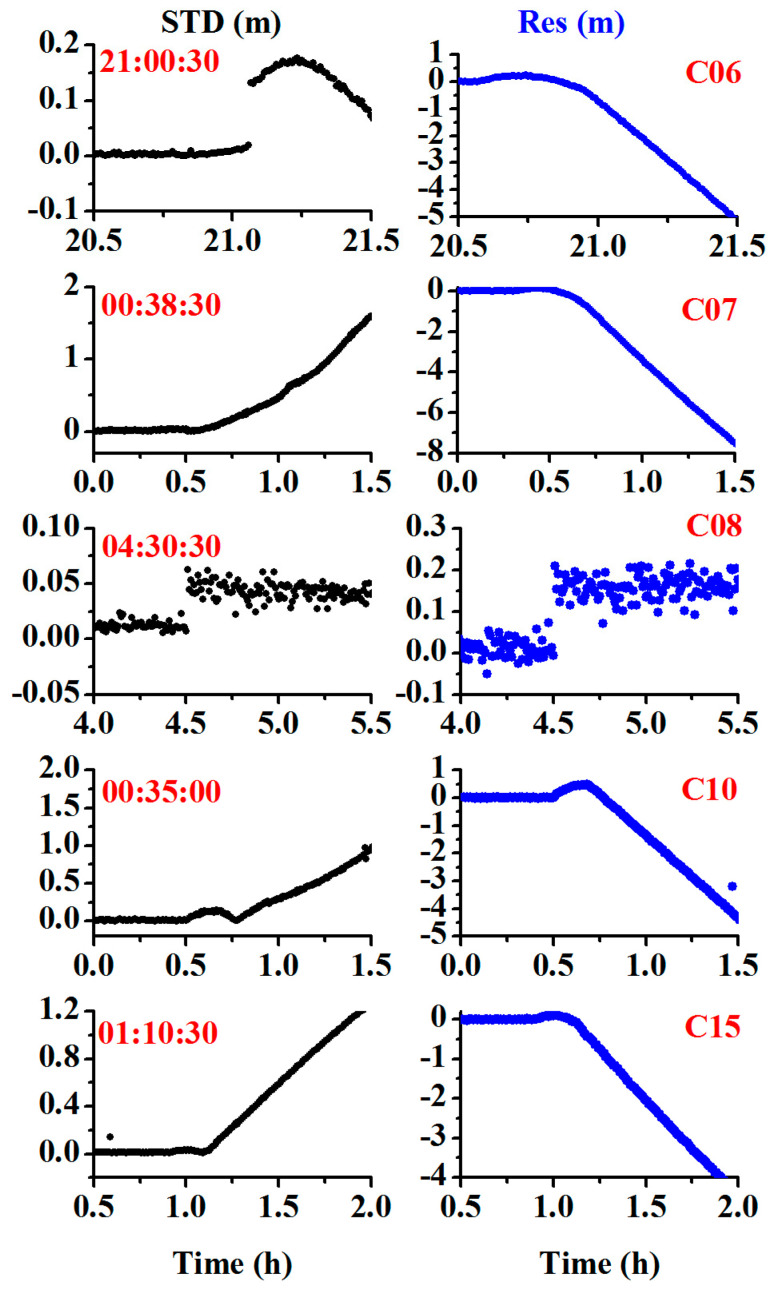
Time series of the STD for the observation residual and array L for the inclined geosynchronous orbit (IGSO) satellites showing orbital maneuver (the left side represents the time discriminant factor, and the right side represents the satellite identification factor).

**Figure 16:**
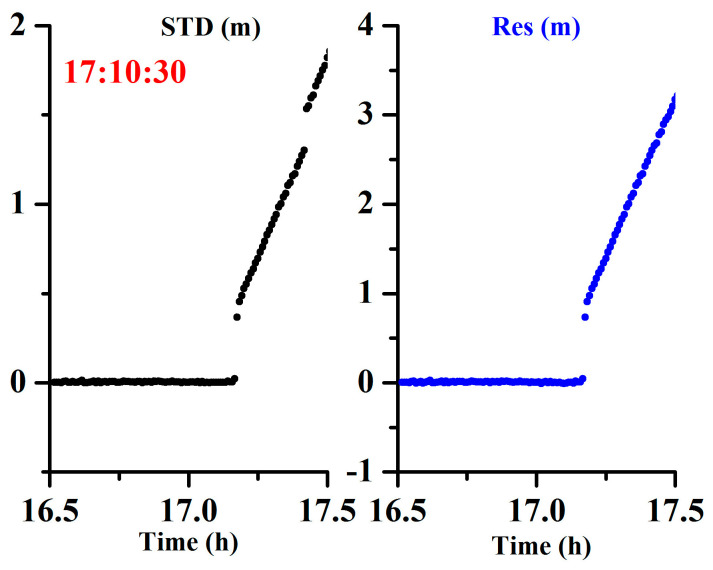
Time series of the STD of the observation residuals and array L for the global positioning system (GPS) medium earth orbit (MEO) satellites showing orbital maneuver (the left side represents the time discriminant factor, and the right side represents the satellite identification factor).

To further validate the performance of the proposed method for orbital maneuvers in different constellations, the BDS GEO and IGSO satellites were tested using the datasets from 2016, and the GPS MEO satellite was tested using datasets from the station BRUN from January 10, 2017. The receiver used was of the TRIMBLE NETR9 type, the antenna used was of the JAVRINGANT_DM SCIS type, and the sampling interval was 30 s. Figure 14, Figure 15 and Figure 16 show the results for the BDS GEO satellites (C01, C02, C03, C04, C05), IGSO satellites (C06, C07, C08, C10, C15), and GPS satellite (G03). Noticeably, the orbital maneuvers for all the satellites were detected due to the abnormality of the time discriminant factor (black color), and the PRN was determined due to the abnormality of the satellite identification factor (blue color).

Table 4 compiles the detected satellites, marked satellites, detected start times, and marked start times. From the table, the time differences between the detected times and the marked times are (1:12:00, 1:25:00, 1:13:00, 1:39:30, 00:50:30, 1:00:30, 0:38:30, 01:30:30, 00:35:00, 01:10:30, 1:10:46) min for (C01, C02, C03, C04, C05, C06, C07, C08, C10, C15, G03), respectively. During these time differences, the observations are still usable as the broadcast ephemeris is marked with unhealthy satellites and the precise ephemeris is not provided. Therefore, the proposed approach can flexibly detect the orbit maneuvers in real time and extend the usable observations.

In addition, the quality of orbits for BDS GEO is very poor, both for broadcast and final orbits [28]. This is because the observations’ geometry between ground stations and satellites does not change and the GEO satellites are in a deep resonance with the Earth’s rotation causing orbital instability, which generates problems in precise positioning and applications [29,30]. However, this does not influence the orbital maneuver detection. As the proposed approach uses the epoch-differenced velocity estimation model, common errors such as orbit residual can be eliminated effectively. Thus, the maneuver detection is not significantly influenced by using either the precise orbit or the broadcast orbit. Moreover, while using the broadcast orbit, the jump in the broadcast orbit boundaries is not recorded as the orbital maneuver because the start time of the orbital maneuver is detected by two conditions: the STD values are greater than the threshold, and the abnormality of the STD values lasts for 5 min. Thus, the jumps in the broadcast orbit boundaries are recorded as outliers.

## 5. Conclusions

This study proposed an approach for the real-time detection of orbital maneuvering using carrier phase observations and broadcast ephemeris data by velocity estimation. It used the STD of the observation residuals to determine the beginning of the maneuver and the satellite-differenced L value of the error equation to determine the PRN of the maneuvering satellite. Using datasets from the four GNSS stations for analysis and validation, the following conclusions can be obtained:

1. When the high-precision carrier phase observation is used, detection by velocity estimation is more sensitive than the SPP approach.

2. The start time and the PRN of the satellite orbital maneuver can be detected accurately in real time using the new approach.

3. The time discriminant factor is consistent and stable across different stations during the non-maneuvering period, and it is very sensitive when the orbital maneuver begins.

4. As the high-rate GNSS exhibits high noise, it is not recommended for use in orbital maneuver detection.

5. The detected start time is usually later than the marked start time from the broadcast ephemeris. In this study, the average time difference is about 67 min for the GEO, IGSO, and MEO satellites, which implies that the proposed method can extend the usable observations by more than 1 h.

In conclusion, the proposed method is processed by a single GNSS receiver, is very flexible for users and useful for IGS analysis centers to precisely determine the orbital maneuvering for precise orbit determination. In future studies, more GNSS data may be tested to further validate the performance of the method.

## Figures and Tables

**Table 1:** Details of the four selected GNSS stations.

Station	Latitude (°)	Long (°)	Height (m)	Receiver	Antenna
WUH1	30.3056	114.2927	71.157	CETC-54-GMR-4016	LEIAR25.R4
BJF1	39.6075	115.8921	75.345	CETC-54-GMR-4016	LEIAR25.R4
POHN	6.9599	158.2101	90.675	SEPT POLARX5	JAVRINGANT_DM
CUT0	-32.0038	115.8947	23.984	TRIMBLE NETR9	TRM59800.00

**Table 2:** Statistics of the STD of the observation residual during the non-maneuvering period (m).

Station	DOY 199			DOY 202		
	Mean	STD	RMS	Mean	STD	RMS
WUH1	0.0082	0.0036	0.0089	0.0077	0.0032	0.0083
BJF1	0.0075	0.0033	0.0082	0.0077	0.0034	0.0084
POHN	0.0044	0.0033	0.0055	0.0049	0.0037	0.0061
CUT0	0.0067	0.0031	0.0074	0.0066	0.0030	0.0072

**Table 3:** Statistics of the L array during the non-maneuver period (m).

Station	DOY 199		DOY 202	
	Average	STD	Average	STD
WUH1	−0.0002	0.018	0.0002	0.021
BJF1	0.0001	0.016	−0.0001	0.024
POHN	−0.0002	0.022	0.0001	0.023
CUT0	0.0003	0.015	−0.0002	0.016

**Table 4:** Results of orbital maneuver detection for the BeiDou Navigation Satellite System (BDS) GEO/IGSO and GPS MEO satellites (unit: hour:minute:second).

PRN	DOY	Detected Time	Marked Time	Difference
C01	009	05:12:00	04:00:00	01:12:00
C02	033	12:25:00	11:00:00	01:25:00
C03	012	08:13:00	07:00:00	01:13:00
C04	088	08:39:30	07:00:00	01:39:30
C05	003	00:50:30	00:00:00	00:50:30
C06	134	21:00:30	20:00:00	01:00:30
C07	138	00:38:30	00:00:00	00:38:30
C08	052	04:30:30	03:00:00	01:30:30
C10	172	00:35:00	00:00:00	00:35:00
C15	229	01:10:30	00:00:00	01:10:30
G03	010	17:10:30	15:59:44	01:10:46
